# A Diagnostic Model Incorporating P50 Sensory Gating and Neuropsychological Tests for Schizophrenia

**DOI:** 10.1371/journal.pone.0057197

**Published:** 2013-02-27

**Authors:** Jia-Chi Shan, Chih-Min Liu, Ming-Jang Chiu, Chen-Chung Liu, Yi-Ling Chien, Tzung-Jeng Hwang, Yi-Ting Lin, Ming H. Hsieh, Fu-Shan Jaw, Hai-Gwo Hwu

**Affiliations:** 1 Department of Psychiatry, Cathay General Hospital, Taipei, Taiwan; 2 Department of Psychiatry, National Taiwan University Hospital, Taipei, Taiwan; 3 Department of Psychiatry, College of Medicine, National Taiwan University, Taipei, Taiwan; 4 Neurobiology and Cognitive Science Center, National Taiwan University, Taipei, Taiwan; 5 Department of Neurology, National Taiwan University Hospital and College of Medicine, National Taiwan University, Taipei, Taiwan; 6 Institute of Biomedical Engineering, National Taiwan University, Taipei, Taiwan; 7 Department of Psychiatry, National Taiwan University Hospital Yun-Lin Branch, Yun-Lin, Taiwan; Chiba University Center for Forensic Mental Health, Japan

## Abstract

**Objectives:**

Endophenotypes in schizophrenia research is a contemporary approach to studying this heterogeneous mental illness, and several candidate neurophysiological markers (e.g. P50 sensory gating) and neuropsychological tests (e.g. Continuous Performance Test (CPT) and Wisconsin Card Sorting Test (WCST)) have been proposed. However, the clinical utility of a single marker appears to be limited. In the present study, we aimed to construct a diagnostic model incorporating P50 sensory gating with other neuropsychological tests in order to improve the clinical utility.

**Methods:**

We recruited clinically stable outpatients meeting DSM-IV criteria of schizophrenia and age- and gender-matched healthy controls. Participants underwent P50 sensory gating experimental sessions and batteries of neuropsychological tests, including CPT, WCST and Wechsler Adult Intelligence Scale Third Edition (WAIS-III).

**Results:**

A total of 106 schizophrenia patients and 74 healthy controls were enrolled. Compared with healthy controls, the patient group had significantly a larger S2 amplitude, and thus poorer P50 gating ratio (gating ratio = S2/S1). In addition, schizophrenia patients had a poorer performance on neuropsychological tests. We then developed a diagnostic model by using multivariable logistic regression analysis to differentiate patients from healthy controls. The final model included the following covariates: abnormal P50 gating (defined as P50 gating ratio >0.4), three subscales derived from the WAIS-III (Arithmetic, Block Design, and Performance IQ), sensitivity index from CPT and smoking status. This model had an adequate accuracy (concordant percentage = 90.4%; *c*-statistic = 0.904; Hosmer-Lemeshow Goodness-of-Fit Test, *p* = 0.64>0.05).

**Conclusion:**

To the best of our knowledge, this is the largest study to date using P50 sensory gating in subjects of Chinese ethnicity and the first to use P50 sensory gating along with other neuropsychological tests to develop a diagnostic model for schizophrenia. Further research to validate the predictive accuracy of this model by applying it on other samples is warranted.

## Introduction

Schizophrenia, a complex psychiatric disorder, clinically encompasses heterogeneous syndromal presentations and pathogenetically involves complicated neurobiological process. The lack of specific corresponding relationships between various phenotypes and neurobiological bases makes it difficult to elucidate the etiology of this mental illness, which is particularly true based upon the phenomenological approach of the contemporary psychiatric classification system. To address this issue, utilizing the concept of endophenotype, i.e. a state-independent intermediate marker, in schizophrenia research has been proposed and widely adopted [1]. A growing body of evidence has suggested several candidate endophenotypes in schizophrenia, including neurophysiological markers (e.g. auditory event-related potentials (ERPs), such as P50, P300 and Mismatch Negativity), and neuropsychological tests (e.g. attention, memory, working memory, and executive function), some of which have been successfully linked to specific genetic components [1]–[3]. Therefore, studying endophenotypes in schizophrenia may lead to a better understanding of this complex illness.

Of neurophysiological markers, P50 sensory gating is regarded to be a candidate endophenotype for schizophrenia [2], [4]. P50, indicating a preattentional electroencephalographic activity, is a positive wave emerging around 50 msec after an auditory stimulus. In a healthy person, the amplitude of P50 wave corresponding to the second click (S2) is significantly smaller than that corresponding to the first (S1). P50 gating ratio, derived from the S2 amplitude divided by S1 amplitude, is the most commonly used index for P50 sensory gating. P50 sensory gating refers to the phenomenon that the central nervous system modulates its sensitivity to incoming stimuli [5]. Regardless of disease state, P50 sensory gating deficit exists in both patients with full-blown schizophrenia and their unaffected relatives with effect sizes of 0.92–1.29 and 0.79, respectively [2]. For schizophrenia patients, there is little evidence for any association between certain symptomatology and P50 gating ratio [6]. Furthermore, P50 sensory gating has been successfully linked to CHRNA7, the gene for alpha-7 subunit of nicotinic receptor, located on chromosome 15q13–14 [7]. Indeed, cigarette smoking has been proved to be able to transiently normalize impaired P50 gating ratio in schizophrenia patients [8]. It has been related to the high prevalence of cigarette smoking in schizophrenia patients, by which, as self-medication, psychiatric symptoms can be alleviated through improving P50 sensory gating deficits [9]. Nevertheless, it remains unclear about the relationship between P50 sensory gating and long-term nicotine exposure. Taken together, these findings support that P50 sensory gating may be qualified as an endophenotype for schizophrenia.

Abundant evidence exists that schizophrenia patients have a poorer performance of neurocognitive function across individual domains (e.g. sustained attention, memory, executive function, working memory, and spatial ability) and global indices (e.g. Performance IQ, Verbal IQ, and Full IQ), some of which have been proposed to be candidate endophenotypes for schizophrenia [2], [3], [10]–[15]. However, it is an issue of grave importance choosing appropriate tools to measure specific neurocognitive performances. Continuous Performance Test (CPT) and Wisconsin Card Sorting Test (WCST) have been widely employed to study neuropsychological deficits in schizophrenia; the former is used to assess sustained attention and the latter executive function. Impaired CPT performance can be found in both schizophrenia patients (effect size: 0.45–3.30) and unaffected relatives (effect size: 0.46–2.97) compared to healthy subjects [2]. Likewise, the effect sizes for WCST in schizophrenia patients and unaffected relatives have been reported to be 0.8–1.0 and 0.26, respectively [15]. Accordingly, both CPT and WCST may be candidate measures of neuropsychological endophenotypes for schizophrenia.

Endophenotypes in schizophrenia, in addition to facilitating research in pathogenesis, may serve another purpose of crucial significance – to refine the classification system, i.e. a clinical purpose [1]. However, the clinical utility of any single candidate endophenotype for schizophrenia to date is limited in contrast to a rapidly growing body of evidence from etiological studies using endophenotypes. This may be because the impairment in either neurophysiological or neuropsychological markers does not exclusively exist in schizophrenia. Taking P50 for example, P50 gating deficits can be observed in bipolar disorder, Huntington’s disease, Alzheimer’s dementia, obsessive-compulsive disorder and attention-deficit/hyperactivity disorder (ADHD) [16]–[21]. Similarly, both CPT and WCST impairments also occur in many psychiatric disorders, such as bipolar disorder, mild cognitive impairment, Alzheimer’s dementia, ADHD and autistic disorder [22]–[29]. One approach to address this issue is to build a diagnostic model by incorporating different markers. Theoretically, the more diversity the markers represent, the more power the model would have in discriminating patients and healthy subjects [30]. Of note, one recent study depicted there existed no associations between P50 gating and neurocognitive domains, including sustained attention, executive function, working memory, verbal learning and memory, visual memory and proceed speed [13].To the present, there have only been few studies focusing on developing models to differentiate schizophrenia from healthy controls or other mental disorders by a combination of different neurophysiological markers, neuropsychological indices, or both [30]–[33].

In the present study, we aimed to construct a diagnostic model for schizophrenia by incorporating P50 sensory gating in combination with other neuropsychological batteries. This work may contribute to the existing literature on P50 sensory gating in subjects of Chinese ethnicity.

## Materials and Methods

### Participants

We enrolled clinically stable outpatients meeting the DSM-IV criteria of schizophrenia and healthy controls of Han Chinese ethnicity with ages ranging from 18 to 65 years. Patients with no medication adjustment and no changes in psychopathology over the past 3 months were defined as clinically stable. Those who suffered from mental retardation, epilepsy or other major brain pathology were excluded. Eligible candidates were evaluated by trained interviewers who administered the Chinese version of the Diagnostic Interview for Genetic Study (DIGS) [34], and further reviewed by two board-certified psychiatrists independently to make psychiatric diagnoses based upon DSM-IV-TR. In cases of discordant opinions, the final diagnoses were determined by a senior psychiatrist. Patients with a diagnosis classified as schizoaffective disorder, bipolar affective disorder, organic mental disorders or substance-related mental disorders were excluded. For controls, additional exclusion criteria included lifetime or current psychiatric diagnoses, and a family history of psychotic disorders.

Demographic characteristics and clinical variables were obtained by interview and medical records, including age, smoking status, amount of tobacco consumption, age at disease onset and antipsychotic medications. Patients’ psychopathology was evaluated using the Positive and Negative Syndrome Scale (PANSS) [35] by treating psychiatrists. The dosage of antipsychotics received was transformed into chlorpromazine (CPZ) equivalent using a standardized method developed by Andreasen et al [36].

This study was approved by the National Taiwan University Hospital Institute Review Board. All participants and/or their guardians provided written informed consent before their participation.

### Electroencephalographic Procedures and Data Processing

Participants who failed to pass audiometric testing, i.e. detecting 40-dB sound pressure level tones at 500, 1000, and 6000 Hz presented to either ear, were excluded. Those who smoked were asked to abstain from smoking for at least 1 hour before sessions [37], [38]. Auditory ERP experiments followed a standard protocol for P50 paired-click paradigm [39]–[41]. The experimental sessions took place in a sound attenuated, electrically shielded booth, with participants lying down in the supine position in a comfortable recliner with their eyes open and focused on a fixation point.

Utilizing Neuroscan STIM system (Compumedics Neuroscan, El Paso, TX, USA), auditory stimuli, which were digitized at 1 kHz with an online band-pass filter at 0.5–100 Hz but no 60-Hz notch filter, were delivered via foam insert earphones binaurally., Corresponding EEG signals were recorded by Neuroscan ACQUIRE system (Compumedics Neuroscan) through a 32-scalp-location (10–20 system) Quik-Cap (Compumedics Neuroscan). The electrode placed on the tip of nose served as reference, and another at Fpz as ground. To monitor blinks and eye movements, four electrodes were placed above and below the left eye and at the outer canthi of both eyes. All electrode impedances were below 5 kΩ.

During the experiment, paired auditory clicks (1 msec, 85 dB) were presented every 8–12 seconds (average: 10 sec), with a 500-msec inter-stimulus interval [42], [43]. A minimum of 120 artifact-free trials were obtained online, in which artifacts were defined as activities exceeding ±100 µV within the time-window of -100–500 msec following stimuli.

All data were processed by researchers blind to the disease status of subjects using Neuroscan Edit 4.3 software (Compumedics Neuroscan) [44]. Tool Command batch processing Language (TCL) was used for semi-automated procedures, beginning with electrooculographic artifact reduction through a built-in pattern-recognition algorithm [45]. The data were epoched for the time window from -100 to 923 msec of the first click, covering both S1 and S2 in the same epoch. Epochs containing activities exceeding ±50 µV were excluded; the remaining epochs, to prevent temporal aliasing, were averaged and digitally bandpass-filtered (10 to 50 Hz) in the frequency domain [46]. Trials with artifacts were manually rejected. By using preset intervals, peaks and preceding troughs were then automatically detected at the Cz electrode [40], [42], [47], [48]. Data with S1 amplitudes less than 0.5µV were removed [44], [49]. When the peak was the largest positive deflection between 45 and 75 msec following the stimuli, the amplitude was the difference between this peak and the preceding negative trough (not earlier than 30 msec post-stimulus). For an S2 response, the activity had to appear within a time window of ±10 msec relative to the latency of the S1 response; otherwise, S2 was regarded as zero [46], [49]. Five parameters were extracted, including S1 latency, S1 amplitude, S2 amplitude, amplitude difference (S1–S2) and P50 gating ratio (S2/S1). For the gating ratio, a maximal value of 2 was applied; those greater than 2 were truncated to 2 as a previously established methodology [49].

### Neuropsychological Assessments

#### Continuous performance test

The paradigm for computerized CPT (i.e. 1–9 task) we adopted in this study has been described in details elsewhere [32], [50]. Briefly, numbers from 0 to 9 were randomly presented on a screen, and the participants were asked to push the button when the number 9 appeared following the number 1. There were two 5-minute sessions: one undegraded and the other 25% degraded. Sensitivity indices, d’ for the undegraded task and mask d’ (md’) for the degraded task, were defined as the ability to discriminate target from non-target stimuli, and were derived from the hit rate and false-alarm rate. These two indicators were presented as adjusted z-scores which were regressed on age, sex and education level according to the data obtained from a healthy community sample [50].

#### Wisconsin card sorting test

The WCST measures executive function. We used a computerized version of the WCST during which participants were asked to match 128 response cards to four stimuli cards according to one of three dimensions, i.e. color, form, and number. Four indicators were extracted and then transformed to adjusted z-sores [32], including: (1) perseverative errors: the number of such kind of errors, indicating perseveration tendency; (2) categories achieved: the number of 10 consecutive correct responses, a measure of overall success; (3) trials to complete first category: the number of trials required to successfully complete the first category, indicating initial conceptualization; and (4) conceptual level response: proportion of 3 or more consecutive correct responses, indicating insight to the correct sorting strategies [51].

#### Chinese version of the wechsler adult intelligence scale third edition

Using the Chinese version of the WAIS-III, we obtained the following indices: (1) Digit Span: a measure of working memory free from distraction; (2) Arithmetic: a measure of working memory ascertained by arithmetic tasks; (3) Digit Symbol - Coding: a measure of processing speed; (4) Block Design: a measure of visuospatial and motor skills; (5) Information: a measure of general knowledge; (6)Working Memory Index: a composite index derived from Digit Span and Arithmetic; (7)Verbal IQ; (8)Performance IQ; and (9) Full IQ [52].

### Statistical Analysis

For demographic characteristics, we compared schizophrenia patients and healthy controls using independent *t* test and chi-square test for continuous and categorical variables, respectively. We also used independent *t* test to examine whether there were any differences between the two groups in terms of P50 parameters and neuropsychological performance.

Moreover, we developed a diagnostic model to best differentiate the schizophrenia patients from the healthy controls by fitting multivariable logistic regression models. Demographic variables, P50 parameters and indicators of neuropsychological test batteries were considered as potential predictive covariates. The stepwise model selection method was used, with significance levels for entry and stay set as 0.15 to identify the important predictors. We used PROC GAM in SAS to explore the relationship between disease status and P50 gating ratio with LOESS smoothing. P50 gating ratio was accordingly dichotomized at a cut-off value of 0.4, obtained by generalized additive models (GAM). We then conducted regression analyses with dichotomized P50 gating ratio again to examine whether continuous or dichotomized P50 gating ratio performed better. Finally, the best model was determined by the metrics of accuracy, i.e. the properties of calibration and discrimination. The former was examined using Hosmer-Lemeshow Goodness-of-Fit test and the latter using *c* statistics. For the purpose of internal validation, the performance of the final model was assessed using bootstrapping technique by which a total of 1000 bootstrap sample sets drawn with replacement of the same size as the observed data was utilized to obtain *c* statistics.

Statistical analyses were conducted using SAS software 9.2 (SAS Institute Inc., Cary, NC, USA). All tests were 2-sided with α = 0.05.

## Results

From October 2006 to September 2008, we recruited 106 schizophrenia patients and 74 healthy controls. There were no differences in age and gender between groups (Table 1). Healthy controls had received more years of education, contained a lower percentage of smokers, and consumed tobacco. For schizophrenia patients, the average age at onset was 23.54±8.10 years and the average duration of illness was 13.67±9.95 years. Psychopathology assessment by PANSS showed a mean total score of 52.54±15.39 (Positive Symptoms subscale: 11.77±4.31; Negative Symptoms subscale: 15.37±6.00; General Symptoms subscale: 25.40±8.18). The average chlorpromazine (CPZ) equivalent dose that patients received was 366.07±232.66 mg per day.

**Table 1:** Demographic data between controls and patients with schizophrenia.

Variable	Control (n = 74)	Schizophrenia (n = 106)	Test statistics^a^	*P*-value^b^
**Gender, n (%)**			0.90	0.343
Male	31 (41.9)	52(49.1)		
Female	43 (58.1)	54(50.9)		
**Age – years, mean(SD)**	36.2 (11.5)	37.2 (10.0)	0.62	0.539
**Education – years, mean(SD)**	15.3 (3.6)	13.8 (2.7)	5.07	<0.001
**Smoking Status**				
Smoker, n (%)	5 (6.8)	18 (17.0)	4.09	0.043
Amount – PPD, mean(SD)	0.05(0.21)	0.16(0.40)	2.46	0.015

SD: standard deviation; PPD: packs per day.

a Independent *t* test for age, years of education and amount of smoking; Chi-square test for gender and smoker.

b *P*-values were 2-sided.

For P50 experiment, the average number of total trials delivered before online rejection of gross artifacts was 170.09±33.85 (patients: 175.44±34.52; controls: 162.43±31.54), and that of trials used for analysis was 111.36±22.70 (patients: 108.09±23.82; controls: 116.03±20.23). Among P50 parameters, the patient group had a significantly larger S2 amplitude and higher P50 gating ratio. However, there were no significant differences in the other P50 parameters, i.e. S1 latency, S1 amplitude and S1–S2 between the two groups (Table 2). With regard to neuropsychological function, schizophrenia patients had a significantly poorer performance than healthy controls across all dimensions, except for the Digit Symbol – Coding subtest of the WAIS-III, in which the difference did not reach statistical significance (Table 3).

**Table 2:** P50 parameters between the controls and patients with schizophrenia.

P50 parameters	Control	Schizophrenia	TestStatistics^a^	*P*-value^b^
	Mean(SD)	Mean(SD)		
S1 latency (msec)	60.62(7.06)	60.94(8.74)	−0.26	0.793
S1 (µV)	2.29(0.87)	2.38(1.17)	−0.59	0.557
S2 (µV)	0.79(0.65)	1.10(0.93)	−2.64	0.009
S2/S1 ratio	0.37(0.37)	0.52(0.46)	−2.25	0.026
S1–S2 (µV)	1.50(0.98)	1.28(1.17)	1.33	0.185

SD: standard deviation.

a The test statistics were obtained by independent *t* tests.

b *P*-values were 2-sided.

**Table 3:** Neuropsychological tests between controls and patients with schizophrenia.

	Control	Schizophrenia	Test Statistics^a^	*P*-value^b^
	Mean(SD)	Mean(SD)		
**CPT**				
d’	−0.03(1.07)	−0.71(1.21)	3.75	<0.001
md’	0.00(0.95)	−0.91(1.36)	5.08	<0.001
**WCST**				
Perseverative errors	−0.16(0.93)	0.75(1.44)	−5.10	<0.001
Categories achieved	0.41(1.02)	−0.48(1.09)	5.44	<0.001
Trials to complete first category	0.06(0.92)	0.46(1.19)	−2.48	0.014
Conceptual level response	0.30(1.02)	−0.57(1.18)	4.97	<0.001
**WAIS-III**				
Arithmetic	12.06(3.11)	8.38(3.30)	7.18	<0.001
Digit span	12.40(3.18)	9.90(4.22)	4.30	<0.001
Information	12.03(2.94)	10.26(3.27)	3.56	<0.001
Digit symbol – coding	11.91 (2.95)	9.96(12.06)	1.49	0.139
Block design	11.99 (2.81)	9.93(6.40)	2.73	0.007
Working memory index	112.5(15.34)	93.14(17.66)	7.28	<0.001
Verbal IQ	113.0(16.28)	95.74(16.76)	6.56	<0.001
Performance IQ	113.5(16.53)	90.58(18.05)	8.29	<0.001
Full IQ	114.1 (15.64)	93.21(16.15)	8.23	<0.001

SD: standard deviation.

a The test statistics were obtained by independent *t* tests.

b *P*-values were 2-sided.

Finally, we fit a multivariable logistic regression model to best differentiate schizophrenia patients from healthy controls, which contained the following covariates: impaired P50 gating (gating ratio >0.4), sensitivity index from CPT (d’), three indices from WAIS-III (Arithmetic, Block Design and Performance IQ), and smoking status. For each variable, the regression coefficient and corresponding odds ratio are listed in the Table 4. This model showed an excellent ability to differentiate schizophrenia patients from healthy controls (*c* statistic = 0.9043; concordant percentage = 90.4%) and fit the observed data well (Hosmer-Lemeshow Goodness-of-Fit test *p* = 0.640). For P50 gating alone and individual neuropsychological tests, the *c* statistics were: 0.578 for impaired P50 gating, 0.675 for d’ from CPT (Hosmer-Lemeshow Goodness-of-Fit test *p* = 0.014), 0.795 for Arithmetic (Hosmer-Lemeshow Goodness-of-Fit test *p* = 0.610), 0.718 for Block Design (Hosmer-Lemeshow Goodness-of-Fit test *p* = 0.058), and 0.824 for PIQ (Hosmer-Lemeshow Goodness-of-Fit test *p* = 0.034). For the model containing three WAIS-III subtests, the *c* statistic was 0.870 (Hosmer-Lemeshow Goodness-of-Fit test *p* = 0.125). When combining CPT and WAIS-III subtests, the *c* statistic of the model was 0.891 (Hosmer-Lemeshow Goodness-of-Fit test *p* = 0.084). Note the majority of the models containing neuropsychological tests fit the observed data poorly, as evidenced by Hosmer-Lemeshow Goodness-of-Fit tests. The inclusion of P50 gating ratio in the final diagnostic model improved both calibration and discrimination. Finally, the internal validation using bootstrapping confirmed the final model having adequate discriminative accuracy (*c* statistic = 0.903±0.024).

**Table 4:** Multivariable logistic regression model for schizophrenia.

Covariate	Coefficient (SE)	Test Statistics^a^	*P*-value^b^	Odds Ratio (95% CI)
Intercept	10.20(2.05)	24.68	<0.001	
Gating	1.00(0.48)	4.28	0.039	2.72(1.05–7.00)
d’	−0.37(0.22)	2.69	0.101	0.69(0.45–1.07)
Arithmetic	−0.32(0.10)	9.91	0.002	0.73(0.60–0.89)
Block Design	0.28(0.14)	3.99	0.046	1.32(1.01–1.74)
Performance IQ	−0.19(0.03)	13.66	0<.001	0.90(0.86–0.95)
Smoke	1.58(.80)	3.88	0.049	4.87(1.01–23.5)

SE: standard error; CI: confidence interval.

Gating is defined as a dichotomous variable: P50 gating ratio greater than 0.4 or not.

Smoke is defined as a dichotomous variable: smoking or not.

^a^ Wald chi-square tests.

^b^ *P*-values were 2-sided.

Multivariable logistic regression model: n  = 160, percentage of concordant pairs  = 90.4%, percentage of discordant pairs  = 9.6%, c statistic  = 0.9043, Hosmer-Lemeshow Goodness-of-Fit test *p*  = .640>.05 (df  = 8).

The estimated probability of having schizophrenia (the predicted value, 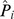) can be obtained by using the following formula:
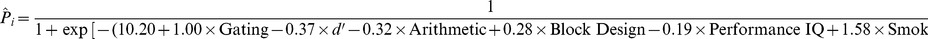

where Gating equals 1 if P50 gating ratio >0.4, and 0 otherwise; Smoke equals 1 if smoking, and 0 otherwise.

## Discussion

To the best of our knowledge, this is the largest study to date using P50 sensory gating in subjects of Chinese ethnicity. [53]. In addition, this study is the first to use P50 sensory gating in combination with other neuropsychological tests in order to develop a diagnostic model to differentiate schizophrenia patients from healthy subjects. As compared with healthy controls, patients with schizophrenia had impaired P50 sensory gating (e.g. P50 gating ratio and S2 amplitude) and neurocognitive functioning (e.g. CPT, WCST and WAIS-III). The best-fit diagnostic model contained impaired P50 gating (P50 gating ratio >0.4), CPT sensitivity index (d’), and three WAIS-III indices (Arithmetic, Block Design and Performance IQ) as predictors, adjusted for smoking status. It was able to satisfactorily identify disease status with adequate calibration and discrimination.

Combining markers representing different neurophysiological and neuropsychological functions appeared to be an effective way to improve diagnostic utility of any single marker. It should be noted that the P50 gating ratio was modeled as a dichotomous variable with a cut-off value of 0.4 in the present diagnostic model. Generally, dichotomizing or categorizing a continuous variable in statistical analysis leads to loss of information, and thus jeopardizes the power. However, in our study, dichotomized P50 gating ratio fit better than keeping it in a continuous form. There may be two reasons for this finding. First, the P50 potential itself inherently suffers from the low signal-to-noise issue [4], [44], [54], [55]. Second, gating ratios greater than 2 were truncated to 2, which was by definition, the maximum value of P50 ratio [49]. That is, in this circumstance, modeling P50 gating ratio as a dichotomous variable may better prevent the association between exposure and outcome from interference by distorted information. In fact, this is in line with previous literature that defined P50 gating deficit as P50 ratio exceeding the cut-off value of 0.4 [56]–[58], which may suggest the use of dichotomous P50 gating for clinical convenience in the future. In addition to P50 gating, four neurocognitive markers used as predictors were also retained in the model. Arithmetic and Block Design, two subsets of the WAIS-III, are measures of working memory and spatial ability, respectively. CPT sensitivity index, d’, measures sustained attention. As previously mentioned, these three neurocognitive functions (i.e. working memory, spatial ability and sustained attention) have been proposed as candidate endophenotypes for schizophrenia [2], [3]. Of the three global intelligence indices of the WAIS, Performance IQ, but not Verbal IQ or Full IQ showed a significant difference between schizophrenia patients and healthy controls, consistent with the findings of a meta-analysis [10]. Regarding smoking, it is well-known that cigarette smoking among schizophrenia patients is highly prevalent [59]. Although the effect of nicotine on P50 gating has been shown to be only short-lived in experimental sessions [37], whether long-term cigarette consumption causes permanent P50-associated neurobiological changes has not to be elucidated. Since smoking is not thought to be a causal factor of schizophrenia, the smoke-status variable in our model served as a confounder for which to be controlled. Taken together, the diagnostic model incorporated markers of biological significance, maximized discrimination power, and took confounding and parsimony into consideration.

With respect to P50 sensory gating, two further implications are worth discussing. Despite not being the first replication study in subjects of Han Chinese ethnicity, this study indeed enrolled the largest sample size of schizophrenia patients (n  = 106) compared to previous studies (n = 50∼80) [53], further supporting the existence of p50 gating deficit in schizophrenia across different populations. Moreover, the role of S2 amplitude should be noted. It is controversial whether deviant P50 gating ratio (S2/S1) results from decreased S1 amplitude or increased S2 amplitude. It has been proposed that S1 reflects registration of novel stimuli and S2 represents habituation of redundant information (“gating out”) [47]. The findings from the present study support the more important role played by S2 than S1 since our patient group had larger S2 amplitude but comparable S1 amplitude when compared with healthy controls. In fact, our results are in line with the findings from one recent meta-analysis which revealed more significant overall effect sizes for S2 (0.65) and P50 gating ratio (0.93) than S1(−0.19), implying the P50 sensory gating deficit in schizophrenia patients is more closely related to impaired ability to filter out irrelevant stimuli [60].

There are several limitations to the present study. First, there may be an issue of generalizability regarding the findings for P50 and neuropsychological tests since the recruited patients consisted of clinically stable outpatients with an average illness duration of 13.67±9.95 years. Second, the possible impact on P50 from antipsychotic medications should be considered. While it is believed that first generation antipsychotics exert no effect on P50 gating, the influence from second generation antipsychotics is inconclusive [6]. However, one recent meta-analysis on Chinese populations found no effect on P50 gating by either first or second generation antipsychotics [53]. Furthermore, we also adjusted for this possible confounding effect by transforming doses of antipsychotics patients took as CPZ equivalent dosage in the regression analyses. Third, since P50 is susceptible to the signal to noise ratio due to its small amplitude, the results obtained from our work might not be generalized to other sensory gating indices, such as N100. Fourth, there lacked a specific measure of memory, another candidate endophenotype for schizophrenia, in our neuropsychological batteries. Therefore, we were unable to examine this important cognitive domain and to determine whether incorporating it could further improve the model performance, which should be addressed by future research. Fifth, in the present study, the possible role and contribution of N100, another candidate endophenotype which also represents sensory gating was not investigated yet, which will be examined and then reported elsewhere in the future. Finally, the transportability of the proposed diagnostic model should be examined by applying it in other samples.

In conclusion, the clinical utility of candidate endophenotypes may be improved by simultaneously integrating several markers into a diagnostic model for schizophrenia. The findings of the present study contribute to the existing literature, especially in terms of expanding and exploring the probable clinical utility of candidate endophenotypes in schizophrenia. Further studies adopting this approach testing more diverse markers, using different populations (e.g. medication-naïve patients, those with early phase psychosis, different ethnic groups), and differentiating schizophrenia from other psychiatric illnesses (e.g. bipolar disorders) are warranted.
